# Sub-Saharan Africa's Mothers, Newborns, and Children: How Many Lives Could Be Saved with Targeted Health Interventions?

**DOI:** 10.1371/journal.pmed.1000295

**Published:** 2010-06-21

**Authors:** Ingrid K. Friberg, Mary V. Kinney, Joy E. Lawn, Kate J. Kerber, M. Oladoyin Odubanjo, Anne-Marie Bergh, Neff Walker, Eva Weissman, Mickey Chopra, Robert E. Black, on behalf of the Science in Action: Saving the lives of Africa's mothers, newborns, and children working group

**Affiliations:** 1 Johns Hopkins Bloomberg School of Public Health, Baltimore, Maryland, United States of America; 2 Saving Newborn Lives/Save the Children, Cape Town, South Africa; 3 The Nigerian Academy of Science, Lagos, Nigeria; 4 MRC Unit for Maternal and Infant Health Care Strategies, University of Pretoria, Pretoria, South Africa; 5 Futures Institute, New York, New York, United States of America; 6 UNICEF, New York, New York, United States of America

## Abstract

As part of the series on maternal, neonatal, and child health in sub-Saharan Africa, Robert Black and colleagues estimated mortality reduction for 42 countries and conclude that the use of local data is needed to prioritize the most effective mix of interventions.

This paper is part of a *PLoS Medicine* series on maternal, neonatal, and child health in Africa.

**Summary Points**

- Sub-Saharan Africa is at a critical point for achieving the Millennium Development Goals for maternal and child survival. Time is short so strategic action is needed now to maximize mortality reduction by 2015.
- We estimated mortality reduction for 42 sub-Saharan African countries if high coverage of MNCH interventions was achieved, using the Lives Saved Tool (LiST). Nearly 4 million African women, newborns, and children need not die each year if already well known interventions reached 90% of families.
- We also undertook a detailed analysis of nine African countries that estimated mortality reductions and additional cost for feasible increases in coverage of selected high-impact MNCH interventions considering three differing health system contexts. It revealed that a 20% coverage increase for selected community-based/outreach interventions would save an estimated 486,000 lives and cost an additional US$1.21 per capita. Increasing the quality of current facility births would save 105,000 lives and cost an additional US$0.54 per capita.
- Functioning health systems require both community-based or outreach services and facility-based care. Maximizing mortality impact for Africa's mothers, newborns, and children depends on using local data to prioritize the most effective mix of interventions, while building a stronger health system.

## Science to Action Gap in Maternal, Neonatal, and Child Health in Sub-Saharan Africa

Over 13,000 mothers, newborns, and children die every day in sub-Saharan Africa—almost nine deaths every minute [1],[2]. Despite being home to just 11% of the world's population, sub-Saharan Africa accounts for half of the global burden of maternal, newborn, and child deaths, two-thirds of global HIV/AIDS deaths, and 90% of global malaria deaths. There are some encouraging signs for maternal, newborn, and child health (MNCH) in Africa with six countries (Botswana, Cape Verde, Eritrea, Malawi, Mauritius, and Seychelles) now on track to achieve Millennium Development Goal (MDG) 4. Attention to and investment in MNCH are increasing [3],[4]. It is critical that this investment is based on priorities that maximize returns, especially given the short time remaining to reach the MDG targets in 2015.

Several papers [5] have reviewed effective health interventions for children [6], newborns [7],[8], and mothers [9],[10]. The continuum of care framework for delivering these key interventions throughout the lifecycle recommends combining single evidence-based interventions into eight MNCH health-service packages at differing health system service delivery levels [11]. Another paper in this series in *PLoS Medicine* on maternal, neonatal, and child health in sub-Saharan Africa summarizes how these interventions can be packaged and shows their current coverage [2].

Low coverage, poor quality, and inequities in the provision of essential MNCH interventions remain a challenge in many sub-Saharan African countries [2],[12]. With an average of only 42% of births occurring in health facilities, there is a *coverage gap* for obstetric care [13]. For births within facilities, a *quality gap* exists and few women and newborns receive the full range of necessary services, with failures to monitor pregnancy and labor, identify complications, and provide life-saving interventions [14]. An *equity gap* exists for skilled birth attendance with coverage 5-fold higher for the least poor versus the poor in many countries [15]. Importantly, however, while identification of such gaps informs national and international health policy makers and program managers where care is lacking, it does not necessarily determine the most effective course of action to save the most lives. Since countries cannot be expected to scale up all essential MNCH interventions simultaneously, prioritization and phasing are required in order to generate success that will lead to increased investment and trust in health systems.

## Context Counts in Selecting Interventions

Sub-Saharan Africa includes 46 countries with substantial variation between and within countries. Local factors must be considered in health planning and prioritization, such as: epidemiology, coverage and utilization of services at all levels of the health system, health system performance (e.g., availability of personnel, equipment and supplies, referral structures, effective supervision), potential platforms for scaling up interventions (e.g., existence of a national cadre of health extension workers, major investments in facility care) as well as funding opportunities and constraints. The diversity of these factors reflects the fact that health systems are complex and include many dimensions. The World Health Organization (WHO) has proposed six essential health system components: governance, financing, human resources, service delivery, logistics and supplies, and information systems [16]. Although there have been many attempts to measure the strength of a health system, such as health expenditures per capita [17] and more complex composite scores [18], an important measure of health system function should reflect health outcomes, ideally mortality. Skilled birth attendance has recently been identified as a useful marker of health system access and equity of services delivery [19],[20], as it is strongly and negatively correlated with maternal and neonatal mortality. Skilled birth attendance is a good predictor of human resource density and demand for health services, both contributing factors to health system performance and quality [15],[20].

In this paper, we estimate the lives that could be saved by scaling up proven health interventions in a variety of health systems, categorized by skilled birth attendance categories, to maximize progress towards MDGs 4 and 5.

## Methods

### Country Selection

We undertook two analyses as follows:

1. For all sub-Saharan African countries with more than 20,000 births per year (42 countries with the exclusion of four with less than 20,000 births [Cape Verde, Mauritius, Sao Tome and Principe, and Seychelles]) we undertook an analysis of lives saved in 2015 for mothers, newborns, and children with 90% coverage of all MNCH interventions.
2. For nine selected sub-Saharan Africa countries we analyzed feasible coverage increases of selected interventions. We selected these nine countries for their range of epidemiology (such as HIV prevalence) and health system contexts and because the academies of sciences in many of these countries are part of the African Science Academies Development Initiative (ASADI), which enabled input from approximately 60 African scientists [1] to the process of intervention selection. The countries are Cameroon, Ethiopia, Ghana, Kenya, Nigeria, Senegal, South Africa, Tanzania, and Uganda, which together account for approximately 50% of sub-Saharan Africa's maternal and child deaths.

Using coverage of skilled attendance at birth, these countries were categorized into three “health system contexts” (Table 1), providing a framework for assessment of priority MNCH interventions in local contexts:

- Low health system context (skilled attendance <30%),
- Middle health system context (skilled attendance 30–60%), and
- High health system context (skilled attendance >60%).

**Table 1:** Summary of the nine example countries split by level of health system context, around the year 2008.

	Low Context (Skilled Attendance <30%)	Middle Context (Skilled Attendance 30–60%)	High Context (Skilled Attendance >60%)	Total
	Ethiopia, Northern Nigeria	Ghana, Kenya, Senegal, Uganda, Tanzania	Cameroon, South Africa, Southern Nigeria	All Nine Countries
**Annual number of births**	6,286,000	5,970,000	4,561,000	16,817,000
**Total number of MNC deaths**	1,079,000	700,000	530,000	2,310,000
**Maternal mortality ratio (deaths per 100,000 live births)**	760	720	833	771
**Neonatal mortality rate (deaths per 1,000 live births)**	49	35	30	38
**Under-five mortality rate (deaths per 1,000 live births**	170	110	110	130
**Skilled birth attendance (%)**	16%	47%	74%	46%
**Facility births (%)**	23%	49%	73%	48%
**Density of health workers (per 1,000)**	0.3	0.7	3	1

Data from Bryce and Requejo, *Countdown to 2015*, 2008 [12] and *State of the World's Children* 2010 [13].

Ethiopia and Northern Nigeria fall in the low skilled birth attendance coverage group (<30%). Nigeria was split into north and south as skilled attendance varies markedly between states—69% in the southern zones and 25% in the northern zones (Table 1). Most of sub-Saharan Africa falls in the middle band (30%–60%), including five of our nine example countries (Ghana, Kenya, Senegal, Uganda, and Tanzania). The higher skilled attendance group (>60%) includes Cameroon, South Africa, and Southern Nigeria.

### Baseline Data

The most recent available estimated rates, numbers, and causes of maternal, neonatal, and child deaths [13],[21]–[24], by country, were used for this exercise as detailed in another paper in the *PLoS Medicine* series [2]. Coverage data are available for many interventions in populous low- and middle-income countries through Demographic and Health Surveys. For some interventions where population-based coverage data are lacking, estimates were made based upon related known coverage indicators, as described in the Lives Saved Tool (LiST) manual [25].

### Intervention Selection, Target Coverage Increases, and Timing

For the analysis for all sub-Saharan African countries, we included all the MNCH interventions in LiST as outlined in another paper [2], building on previous such analysis [7],[26]–[32]. The interventions and their effectiveness sizes as applied are detailed in Table S1. For this analysis, coverage was increased from current levels in 2009 to 90% in 2015 and lives saved in the year 2015 were estimated and summed for all 42 countries and for mothers, newborns, and children.

For the context-specific analysis in the nine selected countries, we considered moderate coverage increases over two years of selected interventions to suit the various health system contexts. Intervention selection was based on: potential mortality impact, affordability, feasibility, and expected effect on equity. Detailed explanations of which interventions were selected for each country and the lives saved and costing results are provided in a previous report [1]. In each health system context, a combination of community/outreach and facility-based targets were chosen.

**Figure 1:**
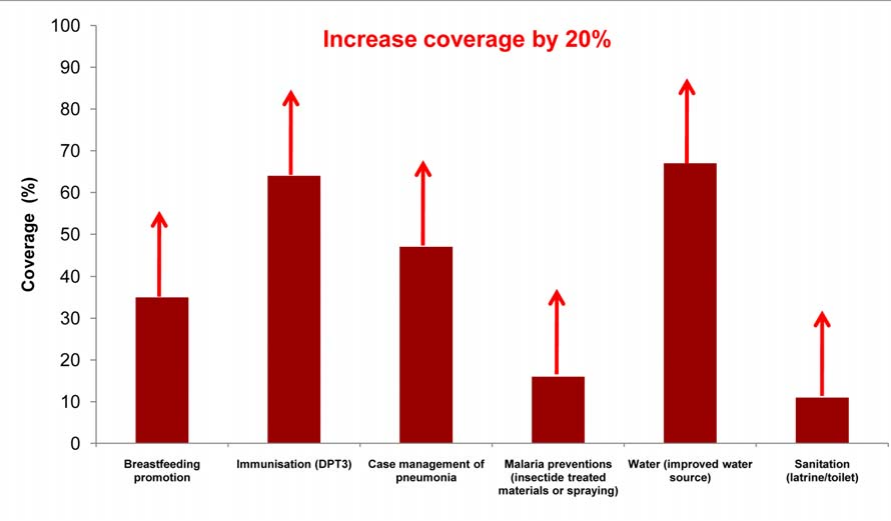
Achievable coverage increases of 20% for outreach/community interventions in Uganda. The figure shows current coverage for some key outreach packages in Uganda with arrows indicating the modeled increase of 20% points within two years. Data from Uganda Demographic Heath Survey, 2006. Some coverages estimated using standard LiST formulas [25].

**Figure 2:**
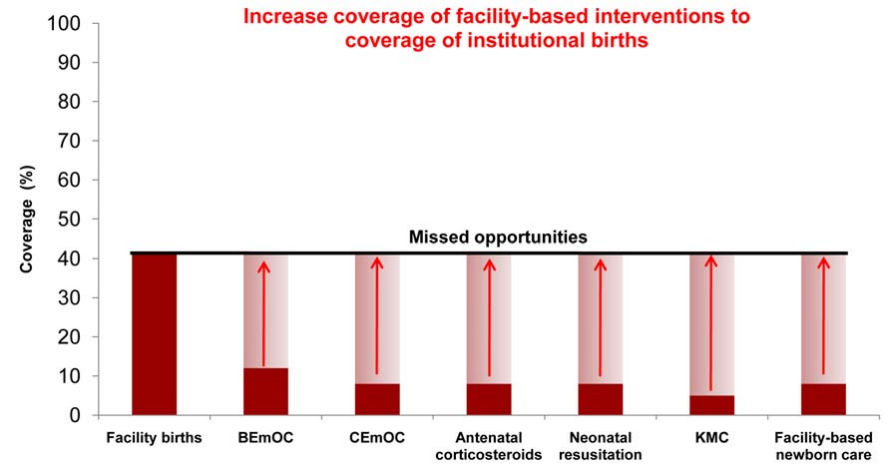
Achievable coverage increases by addressing the quality gap for facility births in Uganda. The figure shows current coverage for some key facility-based MNCH interventions in Uganda with arrows indicating the modeled increase to the current coverage of institutional births within two years. Data from Uganda Demographic Heath Survey, 2006. Some coverages estimated using standard LiST formulas [25].

- *For community/outreach interventions*, we set a target of increasing coverage by 20% within two years, recognizing that in some settings and for some interventions it may be possible to increase coverage more than 20% in two years, while in others this may be challenging. For example, Figure 1 shows current coverage for some key outreach packages in Uganda with arrows indicating the modeled increase for this analysis. The selected interventions for the three health system contexts are shown in table 2. Maternal interventions considered for scale up at the community or outreach level included family planning programs to increase contraceptive prevalence, a rapid and cost effective way to reduce maternal deaths [33]. Tetanus toxoid was considered in low health system contexts to reduce newborn deaths as well as preventive postnatal care, including promotion of healthy practices such as exclusive breastfeeding, clean cord care, and prompt detection and referral for illness, which can be done as an outreach service through home visits delivered by community health workers [34]. For children, preventive practices, including immunizations, vitamin A supplementation, and distribution of insecticide-treated mosquito nets, are essential outreach interventions. Counseling on breastfeeding and complementary feeding, as well as food and vitamin/mineral supplementation, can reduce child mortality and can be delivered at the community-level [29]. Case management of childhood illnesses such as diarrhea, pneumonia, malaria, and measles can occur at the primary care level [35], and is critical in settings with high numbers of child deaths due to infectious diseases. In settings where primary care facilities may be distant, community case management has been shown to be highly effective.
- *For health facility based interventions*, we set a coverage target of increasing *facility-based* MNCH interventions to the current coverage of institutional births, or addressing missed opportunities for these facility births, closing the quality gap. In almost all these countries the coverage of facility births is much higher than the coverage of high impact facility-based interventions required at birth for many women and newborns such as emergency obstetric care, antenatal steroids, neonatal resuscitation, and Kangaroo Mother Care—as demonstrated by Uganda's current coverage levels for facility-based interventions in Figure 2.

**Table 2:** Selected interventions, by health system context and delivery level.

Time	Inteventions	Low Health System	Middle Health System	High Health System
Periconceptional	Contraceptive prevalence rate	O		
Antenatal	Case management during pregnancy			F^a^
	Tetanus toxoid vaccination	O		
Birth	Antenatal corticosteroids for preterm labour		F	F
	Active management of the 3rd stage of labor	F		
	Newborn resuscitation (facility based)		F	F
	Comprehensive emergency obstetric care^a^		F	F
Preventive after birth	Preventive postnatal care	O		F
	Breastfeeding improvements	O	O	O
	Complementary feeding - education only		O	O
	Complementary feeding - supplementation and education		O	O
	Use of improved water source within 30 minutes		O	O
	Use of water connection in the home		O	O
	Improved excreta disposal		O	O
	Hand washing with soap		O	O
	Hygienic disposal of children's stools		O	O
	Insecticide treated materials or indoor residual spraying	O	O	O
	Vitamin A for prevention	O	O	O
	Zinc for prevention		O	O
	Measles vaccine	O	O	O
	Hib vaccine	O	O	O
	Pneumococcal vaccine		O	O
	DPT3 vaccination	O	O	O
Curative after birth	Kangaroo mother care		F	F
	Oral antibiotics for severe infection in neonates	O		
	Injectable antibiotics for severe infection in neonates		O	
	Full supportive care for severe infection in neonates			F
	Oral rehydration salt solution	O	O	O
	Antibiotics for dysentery	O	O	O
	Zinc for treatment	O	O	O
	Case management of pneumonia with oral antibiotics	O	O	O
	Vitamin A for measles treatment	O	O	O
	Antimalarials	O	O	O

F, facility, or increase to total institutional births; O, outreach, or increase by 20%.

a Facility for antenatal coverage is the level of ANC one visit and not the level of facility births like other interventions scaled up to facility level. Facility births are the total of essential care for all women and immediate essential newborn care, basic emergency obstetric care, and comprehensive emergency obstetric care. When scaling up facility births, essential care for all women and immediate essential newborn care and basic emergency obstetric care are scaled down to zero coverage while comprehensive emergency obstetric care is the total of all three, which assumes that all facilities have access to this level of care.

### Modeling Methods

LiST is a new module incorporated into Spectrum based on *The Lancet*'s “Child Survival, Neonatal Survival and Undernutrition” series [5]–[9]. Spectrum is a well-established, free software programme that projects national or subnational demographic change. It is linked to modules for estimating the impact of family planning interventions and AIDS interventions (developed with UNAIDS) [36],[37]. LiST pre-loads national-level health status and mortality data, as well as intervention coverage. The user changes coverage for selected interventions by year—in this exercise, through 2011, using 2009 as the baseline. These changes are linked to cause-specific mortality estimates, resulting in estimates of lives saved for mothers, newborns, and children by intervention and cause per year for that country. The effectiveness values for each intervention come from a standardised review process developed by the Child Health Epidemiology Reference Group (CHERG) with UN partners and using the GRADE criteria to establish which interventions to include and to assess the level of evidence [38]. The detailed review process to estimate cause-specific mortality effectiveness sizes [39], the modelling assumptions in LiST, and many of the specific reviews have recently been published [38],[40]. Additional information on the interventions included and effect sizes applied is available in Table S1.

### Costing Methods

Cost analysis for the interventions was undertaken using the ingredients approach with a focus on additional recurrent cost. Type and amount of drugs, supplies, and personnel time required for each intervention were specified based on standard WHO protocols and expert opinion and then costed using international drug prices from the UNICEF supply catalogue and Management for Sciences Health International Drug Price Indicator, and salary and hospitalization cost data from WHO's CHOICE database (http://www.who.int/choice). Major capital costs such as building of new hospitals were not included as these vary considerably by intervention and by country. For the analyses undertaken here with small increases in community-based interventions or addressing missed opportunities for births already in facilities, the capital costs are not expected to be major.

## Results

### Lives Saved and Costing Results

An achievable scale up of selected *outreach* interventions in the nine selected African countries could avert approximately 22% of maternal, newborn, and child deaths, resulting in nearly half a million lives saved per year (Table 3). On average, the estimated additional cost of increasing these outreach interventions would be approximately US$1.21 per capita; however, this value varies by country.

**Table 3:** Lives saved and costing results for MNCH in the nine countries.

Step	Scale-Up	*Percent of deaths averted* (lives saved)	Additional cost
Step 1	Achievable scale-up of selected MNCH outreach interventions by increasing coverage by 20%*	*22% of MNC deaths averted* (486,000 in all 9 countries†)	US$ 1.21 per capita
Step 2	Achievable scale-up of selected maternal and newborn facility-based interventions by ensuring all facility births received the interventions	*26% of MN deaths averted in 7 countries* (105,000 in the selected middle and high context countries‡)	US$ 0.54 per capita
Step 3	Address specific disease problems, for example HIV/AIDS	Situation dependent	Situation dependent
Step 4	Targeted health system strengthening to reach high coverage of all essential MNCH interventions	*85% of MNC deaths averted* (3.98 million in 42 sub-Saharan African countries)	Not calculated

*Specific interventions included in the analysis are available in Table 2 and Table S1. Additional costing results are available in Table S2.

**†:** The nine selected countries are Cameroon, Ethiopia, Ghana, Kenya, Nigeria, Senegal, South Africa, Tanzania, and Uganda.

**‡:** Step 2 percent based only on maternal and neonatal deaths averted in the middle and high impact countries; Ethiopia and Northern Nigeria are excluded.

*Outreach* interventions for mothers and newborns were only considered for countries with low health system contexts, whereas *outreach* interventions for children were considered in all three health system contexts. Increased use of modern contraceptives could avert a quarter of maternal deaths each year in these two places and would only cost an additional US$0.17 per capita. High-impact newborn outreach interventions, from Table 2, if scaled up by 20%, could save nearly 24,000 lives in Ethiopia and Northern Nigeria at an estimated average cost of US$0.03 per capita. Child survival would benefit substantially by expanding coverage of preventive and curative interventions that can be delivered at the community level or through health facility outreach. The results indicate that nearly a half million child lives could be saved each year at an additional cost of US$1.13 per capita in the nine example countries.

*Facility-based* interventions for maternal and newborn health were scaled up to the current level of institutional births only in countries with middle and high health system contexts. The results of closing this quality gap for some interventions (Table 2) for current for facility-based births indicate that an estimated 26% of maternal and newborn deaths could be averted in the selected African countries, resulting in nearly 105,000 lives saved per year (Table 3). On average, the estimated additional cost of increasing coverage would be approximately US$0.54 per capita.

For mothers and newborns, if deliveries already occurring in facilities had access to CEmOC in middle and high health system contexts, 13,000 mothers could be saved each year—or 17% of maternal deaths—and 29,000 newborn lives could be saved, or 9% of neonatal deaths. The estimated additional cost for this would be approximately US$0.20 per capita, making quality improvement of facility care cost-effective for mothers and newborns. Ensuring that all babies born in facilities receive key specific *facility-based* interventions (Table 2) for neonates in middle- and high-performing health systems could save an additional 90,000 newborns each year, preventing another 28% of newborn deaths, at an estimated cost of US$0.33 per capita.

## Implications

With policy attention increasingly focused on the link between MDGs 4 and 5, there is demand for and value in showing results with benefit for multiple outcomes within MNCH. The results presented here derive from the first modeling exercise to show a combination of maternal, newborn, and child lives saved in sub-Saharan Africa. This study considers moderate coverage increases for *community*/*outreach* interventions and addresses the quality gap for the 42% of births already occurring in facilities by increasing facility-based interventions in an attempt to illustrate possible steps for African health systems, wherever the starting point, to achieve meaningful mortality change in the short term while building stronger health systems.

We focus on the prioritization of high-impact interventions to implement within health systems, rather than the process of implementation, which is also critical. The supply side investments in human resources, medicine logistics, and so on, also involve addressing demand-side barriers, including a range of sociocultural factors around accessing care, distance to health facilities, and direct and indirect costs of health care. Reducing all such barriers that prevent pregnant women from going to facilities may require innovative approaches, such as emergency funds, transport schemes, and maternity waiting homes [33].

## Strengthening Health Systems Step-by-Step

There is a plethora of literature on health system strengthening, but one common thread recommends starting with simple approaches and using those to build human resources and strengthen already existing programs. This has been called the “diagonal” approach, and argues that “vertical” strategies, which focus on interventions for specific diseases, can be used to strengthen “horizontal” strategies, which are the structures and functions of the health system [34]. Our analysis illustrates this by using local data and lives saved analyses to inform which health system priorities are likely to be feasible initial steps, and ultimately strengthen the MNCH components of health systems.

### Step 1: Select a Limited Number of High-Impact Outreach Interventions and Increase Coverage by a Feasible Amount

In health system settings with low levels of current access and utilization of health care facilities, large-scale public health interventions delivered through outreach channels are more feasible to increase initially and can ensure access of the poor to basic services while health facilities are being strengthened and services made more equitable. Countries in these settings experience critical constraints in the delivery of complex packages especially with regard to management capacity of supplies and logistics.

Many African countries continue to experience a shortage of contraceptive supplies despite the low cost [41]. Family planning uptake is also dependent on the empowerment of women and shifting social norms regarding family size. Preventive postnatal care and increased exclusive breastfeeding may be achieved through community mobilization and media campaigns. But early postnatal contacts, such as home visits, are more effective in reaching recently delivered mothers in order to promote healthy practices, identify illness, and link the mother and baby with the health facility [34]. In settings where referral and access to facilities is weak, case management may be done at lower levels. For example, Ethiopia has just mandated community case management of pneumonia to be implemented by the 30,000 newly trained and deployed Health Extension Workers.

Public health interventions such as immunization, that do not require schedulable services, are more amenable to relatively rapid improvements, and are already at high coverage levels in many countries. However, there are still constraints, such as maintaining the cold chain for vaccines and other critical supply management issues that hamper progress. While increasing the supply of services at the lowest levels, conditional cash transfers and other incentives may also be used to increase demand, especially for the poorest families.

### Step 2: Address Missed Opportunities for Births in Health Facilities

Countries with greater access to and utilization of health care facilities can seize opportunities to ensure that all mothers, newborns, and children cared for in health facilities actually receive the highest level of care possible. Since the cost of care during pregnancy and childbirth is one of the main contributors to delays in accessing care, restructuring the health system to provide low-cost public health services or abolishing user fees are proven strategies for increasing the number of facility births, as experienced in Ghana and South Africa [42],[43]. To address delays in receiving care within facilities, often related to gaps in quality of care, accountability mechanisms such as mortality audits can be used [44].

For the most part, facility-based maternal and newborn interventions are feasible additions to already existing services, such as ensuring that every birth attendant can resuscitate a nonbreathing newborn [45]. Functional logistics management and competency-based training for health workers are required to maintain coverage and increase quality.

### Step 3: Identify and Address Specific Disease Problems

Strengthening health systems also requires consideration of the local health burden and other locally specific challenges, e.g., malaria, HIV/AIDS, conflict, complex emergencies, and inequity for specific groups. The case of HIV/AIDS in South Africa demonstrates how context affects MNCH and shapes the responsiveness of the health system. South Africa has about 300,000 HIV-infected mothers giving birth to infants every year with HIV/AIDS, contributing to 57% of all child deaths and more than 80% of child deaths after the first month of life. The results of this LiST analysis suggests that if South Africa scaled up interventions for prevention of mother-to-child transmission of HIV (PMTCT) with appropriate feeding choices to cover 95% of mothers and newborns, over 37,000 children could be saved each year [32]. Strategic investments in neonatal health packages could save an additional 12,000 lives a year and foster integration with existing HIV/AIDS services. Yet, gaps remain and limit this potential improvement. For example, coverage of exclusive breastfeeding is below 10%, reflecting the challenges of conflicting and changing messages of optimal feeding within HIV education and counselling. South Africa has the potential to reverse trends of increasing child mortality and even shift to being on track to achieving MDG 4 with rapid scale up of PMTCT, a context-specific solution.

### Step 4: Strengthen the Health System to Reach High Coverage of All Essential MNCH Interventions

High-impact opportunities for MNCH, when scaled up to coverage levels achievable in the short-term, could save hundreds of thousands of lives in sub-Saharan Africa. However, the overall goal is to reach high coverage of all essential MNCH interventions. If 90% of Africa's families could receive effective and consistent implementation of essential MNCH interventions by 2015, nearly 4 million maternal, neonatal, and child deaths could be prevented each year—an 85% reduction in mortality [1]. Countries in low health system contexts starting at a lower level of coverage have greater potential for rapid increases. However, even in countries with high skilled attendance and better health systems, almost one million lives could be saved if MNCH interventions reached all those who need them (Table S1). This aspirational target suggests that most mothers, newborns, and children need not die in the region and should serve as a wake-up call to governments, health policy planners, and development partners to strategically assess their current MNCH status, use national data to identify high-impact interventions, set achievable coverage targets in the short- and long-term, and effectively implement strategies through proven health-service packages.

## Conclusions

There are three main conclusions to draw from this analysis:

### 1. Modest Increases in Selected Outreach Interventions Can Save Lives Now

Much can be done at community level for children through improving nutrition, providing vaccinations, and preventing and treating malaria, diarrhea, and pneumonia. Community level provision of contraceptives can have a significant impact on maternal mortality. These, with other community-based interventions, can also reduce maternal and neonatal deaths.

### 2. Addressing Missed Opportunities for Births Already Occurring in Health Facilities Can Also Save Maternal and Newborn Lives Now

Strengthening existing programs within health facilities could prevent many deaths, even without high-tech equipment and supplies [15]. Many newborn deaths could be prevented with facility-based interventions such as neonatal resuscitation, hygienic practices, and thermal care around the time of birth for all neonates, as well as antenatal steroids and Kangaroo Mother Care for preterm babies. Since more than half of maternal deaths in sub-Saharan Africa are due to obstetric complications, it is critical to ensure that women with life-threatening complications can access the emergency obstetric care that can save their life and that of their baby.

### 3. Consideration of Local Data and Different Health System Settings Is Necessary to Identify High-Impact, Short-Term Opportunities That Are Appropriate and Feasible for Given Health System Environments

While much is known about interventions that can save lives, there are still unanswered questions regarding the “who” and “how to” around optimal service delivery strategies, providing care to families close to home, and reaching hard-to-serve populations. There is a gap in the use of local and representative data to inform policy, practice, and research priorities. There is also an urgent need to strengthen and disseminate existing tools such as LiST to assist governments and policy makers, including at the local level, in setting priorities and targets. Once evidence-based priority interventions are identified, it is necessary to link these interventions to policy as well as to address implementation challenges. There are a number of immediate opportunities available even in the lowest resource settings; however, a shortage of qualified health workers is a major constraint for improving essential health care in sub-Saharan Africa [46]. This is true both in direct service provision as well as in lack of public health champions to lead the way towards policy change [47]. More health systems research is needed on optimum delivery strategies for specific interventions and health care packages given existing constraints, and on how to increase coverage with existing packages within individual countries [19].

Despite often negative publicity, some African countries are making progress towards saving the lives of mothers, newborns, and children. Even more lives can be saved if countries use local data to identify priority interventions and increase coverage and quality in the short term. Local and national governments and policy makers should be encouraged to use science to inform effective action to save the lives of sub-Saharan Africa's mothers, newborns, and children.

## Supporting Information

Table S1 Detailed information on LiST including effect sizes.
(0.11 MB DOC)

Table S2 Further detail about costing exercise.
(0.03 MB DOC)

## Abbreviations

BEmOC: Basic Emergency Obstetric Care
CEmOC: Comprehensive Emergency Obstetric Care
LiST: Lives Saved Tool
MDGs: Millennium Development Goals
MMR: Maternal Mortality Rate
MNCH: Maternal, Newborn and Child Heath
NMR: Neonatal Mortality Rate
PMTCT: Prevention of Mother-To-Child Transmission of HIV
U5MR: under-5 mortality rate
WHO: World Health Organization
