# Supplementary material for: Sub-Saharan Africa's Mothers, Newborns, and Children: How Many Lives Could Be Saved with Targeted Health Interventions?
Source: PLoS Med. 2010 Jun 21;7(6):e1000295. doi: 10.1371/journal.pmed.1000295 (PMC2888572; doi:10.1371/journal.pmed.1000295)
Supplement: Table S1 — Detailed information on LiST including effect sizes. (0.11 MB DOC) [file pmed.1000295.s001.doc]

**Supplemental Table S1. Details on *LiST* Effect sizes in version 3.40**

Effect Sizes related to Maternal Causes of Death that were used

| Cause of Death | Intervention | Effect |  | Cause of Death | Intervention | Effect |
| --- | --- | --- | --- | --- | --- | --- |
| Hypertensive diseases | Comprehensive Emergency Obstetric Care | .95 |  | Obstructed labor | Basic Emergency Obstetric Care | .08 |
|  | Calcium supplementation | .20 |  |  | Comprehensive Emergency Obstetric Care | .99 |
|  | Comprehensive Emergency Obstetric Care | .96 |  | Ectopic pregnancy | Ectopic pregnancy case management (BEmOC level) | .3 |
| Infections | Antibiotics for pPRoM | .26  (.1) |  |  | Ectopic pregnancy case management (CEmOC level) | .9 |
|  | Clean practices and immediate essential newborn care (home) | .1  (.5) |  | Malaria | IPTp | .4 |
| Postpartum Hemorrhage | Essential care for all women and immediate essential newborn care (facility) | .1  (.5) |  | Other indirect causes | Tetanus toxoid immunization | .98 (.005) |
|  | Basic Emergency Obstetric Care | .5 |  | Antepartum Hemorrhage | Basic Emergency Obstetric Care | .2 |
|  | Comprehensive Emergency Obstetric Care | .7 |  |  | Comprehensive Emergency Obstetric Care | .8 |
|  | Active management of the third state of labor | .27 |  | Note: All affected fractions are equal to 1 unless otherwise stated. All numbers in parentheses are the relevant affected fractions | | |
|  | Basic Emergency Obstetric Care | .65 |  |  |  |  |

**Effect Sizes related to Neonatal Causes of Death**

| Cause of Death | Intervention | Effect |  | Cause of Death | Intervention | Effect |
| --- | --- | --- | --- | --- | --- | --- |
| Diarrhea | ORS | 0.93 |  | Prematurity | Antenatal corticosteroids for preterm labor | 0.53 |
| Sepsis Pneumonia | Syphilis detection and treatment | 0.025 |  |  | Antibiotics for pPRoM | 0.12 |
|  | Antibiotics for pPRoM | 0.08 |  |  | Essential care for all women and immediate essential newborn care | 0.10 |
|  | Essential care for all women and immediate essential newborn care | 0.25 |  |  | Basic emergency obstetric care | 0.10 |
|  | Basic emergency obstetric care | 0.25 |  |  | Comprehensive emergency obstetric care | 0.10 |
|  | Comprehensive emergency obstetric care | 0.25 |  |  | Neonatal resuscitation (institutional) | 0.10 |
|  | Clean practices and immediate essential newborn care (home) | 0.20 |  |  | Neonatal resuscitation (home) | 0.05 |
|  | Preventive postnatal care (healthy practices & illness detection) | 0.31 |  |  | Preventive postnatal care (healthy practices & illness detection) | 0.35 |
|  | Oral antibiotic case management of severe infection | 0.42 |  |  | Kangaroo mother care | 0.51 |
|  | Injectable antibiotic case management of severe infection | 0.68 |  |  | Case management of severe illness with full supportive care | 0.28 |
|  | Case management of severe infection with full supportive care | 0.83 |  | Tetanus | Tetanus toxoid | 0.94 |
| Asphyxia | Essential care for all women and immediate essential newborn care | 0.25 |  |  | Essential care for all women and immediate essential newborn care | 0.36 |
|  | Basic emergency obstetric care | 0.40 |  |  | Basic emergency obstetric care | 0.36 |
|  | Comprehensive emergency obstetric care | 0.80 |  |  | Comprehensive emergency obstetric care | 0.36 |
|  | Neonatal resuscitation (institutional) | 0.30 |  |  | Clean practices and immediate essential newborn care (home) | 0.30 |
|  | Neonatal resuscitation (home) | 0.20 |  | Congenital anomalies | Periconceptual Folic Acid | 0.35 |
|  | Case management of severe infection with full supportive care | 0.05 |  | Other | Case management of serious neonatal illness | 0.10 |

Effect Sizes related to Childhood Causes of Death that were used

| Cause of Death | Intervention | 1-6 months | | 6-12 months | | 12-23 months | | 24-59 months | |
| --- | --- | --- | --- | --- | --- | --- | --- | --- | --- |
|  |  | Effect | AF | Effect | AF | Effect | AF | Effect | AF |
| Diarrhea | Use of improved water source within 30 minutes | .17 | 1 | .17 | 1 | .17 | 1 | .17 | 1 |
|  | Use of water connection in the home | .69 | 1 | .69 | 1 | .69 | 1 | .69 | 1 |
|  | Improved excreta disposal (latrine/toilet) | .36 | 1 | .36 | 1 | .36 | 1 | .36 | 1 |
|  | Hand washing with soap | .48 | 1 | .48 | 1 | .48 | 1 | .48 | 1 |
|  | Hygienic disposal of children's stool | .20 | 1 | .20 | 1 | .20 | 1 | .20 | 1 |
|  | Vitamin A for prevention | 0 | 1 | 0.31 | 1 | 0.31 | 1 | 0.31 | 1 |
|  | Zinc for prevention | 0 | 1 | 0.13 | 1 | 0.13 | 1 | 0.13 | 1 |
|  | Rotavirus vaccine | .74 | 0.39 | 0.74 | 0.39 | 0.74 | 0.39 | 0.74 | 0.39 |
|  | ORS | 0.93 | 0.95 | 0.93 | 0.95 | 0.93 | 0.95 | 0.93 | 0.95 |
|  | Antibiotics for dysentery | 0.99 | 0.05 | 0.99 | 0.05 | 0.99 | 0.05 | 0.99 | 0.05 |
|  | Zinc for treatment | 0.23 | 1 | 0.23 | 1 | 0.23 | 1 | 0.23 | 1 |
| Pneumonia | Zinc for prevention | 0 | 1 | 0.15 | 1 | 0.15 | 1 | 0.15 | 1 |
|  | Hib vaccine | 0.18 | 1 | 0.18 | 1 | 0.18 | 1 | 0 | 1 |
|  | Pneumococcal vaccine | 0.24 | 1 | 0.24 | 1 | 0.24 | 1 | 0.24 | 1 |
|  | DPT vaccination | 0.1 | 1 | 0.1 | 1 | 0.1 | 1 | 0.1 | 1 |
|  | Case management of pneumonia (oral antibiotics) | 0.7 | 1 | 0.7 | 1 | 0.7 | 1 | 0.7 | 1 |
| Measles | Measles vaccine | .85 | 1 | 0.85 | 1 | 0.85 | 1 | 0.85 | 1 |
|  | Vitamin A for measles treatment | 0.62 | 1 | 0.62 | 1 | 0.62 | 1 | 0.62 | 1 |
| Malaria | Insecticide treated materials/indoor residual spraying | 0.55 | 1 | 0.55 | 1 | 0.55 | 1 | 0.55 | 1 |
|  | Antimalarials | 0.84 | 1 | 0.84 | 1 | 0.84 | 1 | 0.84 | 1 |

Note: Interventions which were not part of this exercise are not listed on this table. For breastfeeding information see The Lancet Undernutrition series. For effects relating to HIV – see AIM Manual for Spectrum.

**References and systematic reviews related to the *Lives Save Tool*:**

1. Stover J, McKinnon R, Winfrey B. (2010) Spectrum: A model platform for linking of maternal and child survival interventions with AIDS, family planning and demographic projections. IJE. In press
2. Boschi-Pinto C, Young M, Black RE. (2010) The Child Health Epidemiology Reference Group Reviews of the Effectiveness of Interventions to Reduce Maternal, Neonatal and Child Mortality. IJE. In press
3. Walker N, Fischer-Walker C, Bryce J, Bahl R, Cousens S, writing for the CHERG Review Groups on Intervention Effects. (2010) Standards for CHERG Reviews of Intervention Effects on Child Survival. IJE. In Press.
4. Traa B, Fischer-Walker C, Munos M, Black RE. (2010) Antibiotics for the Treatment of Dysentery in Children. IJE. In Press.
5. Munos M, Fischer-Walker C, Black RE. (2010) The Effect of Oral Rehydration Solution and Recommended Home Fluids on Diarrhea Mortality. IJE. In Press
6. Munos M, Fischer-Walker C, Black RE. (2010) The effect of rotavirus vaccine on diarrhea mortality. IJE. In Press.
7. Fischer-Walker C, Black RE. (2010) Zinc for the treatment of diarrhea: Effect on diarrhea morbidity, mortality, and incidence of future episodes. IJE. In Press.
8. Theodoratou E, Al-Jilaihaw S, Woodward F, Ferguson J, Jhass A, et al. (2010) The effect of case management on childhood pneumonia mortality in developing countries. IJE. In Press.
9. Eisele TP, Larsen D, Steketee RW. (2010) Protective efficacy of interventions for preventing malaria mortality in children in *Plasmodium falciparum* endemic areas. IJE. In Press.
10. Sudfeld CR, Navar AM, Halsey NA. (2010) Effectiveness of Measles Vaccination and Vitamin A Treatment. IJE. In Press.
11. Theodoratou E, Johnson S, Jhass A, Madhi SA, Clark A, et al. (2010) The effect of *H*aemophilus *influenzae* type b and pneumococcal conjugate vaccines on childhood pneumonia incidence, severe morbidity and mortality. IJE. In Press.
12. Cairncross S, Hunt C, Boisson S, Bostoen K, Curtis V, et al. (2010) Water, sanitation and hygiene for the prevention of diarrhoea. IJE. In Press.

**Comments on Limitations of *LIST***

Uncertainty exists around all data, including both *LiST* data inputs and outcomes. Interventions already at high coverage levels result in few additional lives saved which may be misunderstood. For example, immunisation coverage is high in South Africa so few additional lives are saved by moving from over 90% to a target of 95% coverage. However, if investment stopped and coverage fell then mortality would rise.

In addition, the effect sizes which are used in LiST are intended to be effectiveness sizes. In some cases, this quality of data was not available and alternatively efficacy data was used when available. Please read the review of hte GRADE methodology as applied to the LiST interventions for further clarification.
