# Supplementary material for: Sub-Saharan Africa's Mothers, Newborns, and Children: How Many Lives Could Be Saved with Targeted Health Interventions?
Source: PLoS Med. 2010 Jun 21;7(6):e1000295. doi: 10.1371/journal.pmed.1000295 (PMC2888572; doi:10.1371/journal.pmed.1000295)
Supplement: Table S2 — Further detail about costing exercise. (0.03 MB DOC) [file pmed.1000295.s002.doc]

Supplemental web table 2. Total additional costs required by country for each package of interventions.

|  | Low health system context | | Middle health system context | | | | | High Health system context | | |
| --- | --- | --- | --- | --- | --- | --- | --- | --- | --- | --- |
|  | Ethiopia | Northern Nigeria | Ghana | Kenya | Senegal | Tanzania | Uganda | Cameroon | Southern Nigeria | South Africa |
| Maternal | 12,664,321 | 10,769,269 | 6,459,547 | 11,068,694 | 2,254,579 | 11,127,389 | 11,027,042 | 2,773,998 | 5,417,314 | 5,858,429 |
| Neonatal | 1,110,645 | 2,679,817 | 8,116,509 | 17,968,481 | 11,234,010 | 12,682,026 | 19,930,023 | 5,316,939 | 38,557,697 | 61,899,413 |
| Child | 27,114,345 | 51,032,859 | 30,297,217 | 54,669,672 | 22,631,031 | 64,614,940 | 58,797,596 | 31,974,263 | 92,095,391 | 36,416,108 |
